# A dataset of cold-water coral distribution records

**DOI:** 10.1016/j.dib.2023.109223

**Published:** 2023-05-11

**Authors:** Viktória Balogh, Eliza Fragkopoulou, Ester A. Serrão, Jorge Assis

**Affiliations:** a Center of Marine Sciences (CCMAR-CIMAR), University of the Algarve, 8005-139 Faro, Portugal; b Faculty of Bioscience and Aquaculture, Nord Universitet, Postboks 1490, Bodø, Norway

**Keywords:** Marine ecosystem, Marine biodiversity data, Biogeographic information, Occurrence records

## Abstract

Species distribution data are key for monitoring present and future biodiversity patterns and informing conservation and management strategies. Large biodiversity information facilities often contain spatial and taxonomic errors that reduce the quality of the provided data. Moreover, datasets are frequently shared in varying formats, inhibiting proper integration and interoperability. Here, we provide a quality-controlled dataset of the diversity and distribution of cold-water corals, which provide key ecosystem services and are considered vulnerable to human activities and climate change effects. We use the common term cold-water corals to refer to species of the orders Alcyonacea, Antipatharia, Pennatulacea, Scleractinia, Zoantharia of the subphylum Anthozoa, and order Anthoathecata of the class Hydrozoa. Distribution records were collated from multiple sources, standardized using the Darwin Core Standard, dereplicated, taxonomically corrected and flagged for potential vertical and geographic distribution errors based on peer-reviewed published literature and expert consulting. This resulted in 817,559 quality-controlled records of 1,170 accepted species of cold-water corals, openly available under the FAIR principle of Findability, Accessibility, Interoperability and Reusability of data. The dataset represents the most updated baseline for the global cold-water coral diversity, and it can be used by the broad scientific community to provide insights into biodiversity patterns and their drivers, identify regions of high biodiversity and endemicity, and project potential redistribution under future climate change. It can also be used by managers and stakeholders to guide biodiversity conservation and prioritization actions against biodiversity loss.

**Specifications Table**

**Table:** 

Subject	Biodiversity
Specific subject area	Biodiversity information, Biogeography, Marine ecological conservation and management, Climate change assessments.
Type of data	Table Chart Graph Figure
How the data were acquired	Collection of occurrence records from major biodiversity information facilities and peer-reviewed scientific literature. Occurrence data were processed using the R statistical computing software, version 2022.
Data format	Excel files (Raw and Filtered)
Description of data collection	Georeferenced occurrence records of cold-water corals (orders Alcyonacea, Antipatharia, Pennatulacea, Scleractinia, Zoantharia of the subphylum Anthozoa, and order Anthoathecata of the class Hydrozoa) were collated from biodiversity information facilities and peer-reviewed scientific literature. Data were standardized with Darwin Core Standard, dereplicated, cross-checked for taxonomic issues and errors in distribution records (records on land, outside of known depth ranges and geographic distributions) were flagged for removal based on published information, databases of biological traits and expert consulting.
Data source location	Institution: CCMAR- Center of Marine Sciences, City/Town/Region: Faro, Algarve, Country: Portugal. A dataset of cold-water coral diversity compiled from the following biodiversity information facilities: (1) AquaMaps (http://aquamaps.org); (2) European Marine Observation and Data Network (https://emodnet.ec.europa.eu); (3) Global Biodiversity Information Facility (https://www.gbif.org); (4) Deep Sea Coral Base, National Oceanic and Atmospheric Administration (https://deepseacoraldata.noaa.gov/data); (5) Ocean Biodiversity Information System (https://obis.org); (6) The Coral Trait Database (https://coraltraits.org); (7) Vulnerable Marine EcoSystems Dataset, International Council for the Exploration of the Sea (https://vme.ices.dk/download.aspx).
Data accessibility	Repository name: Figshare Data identification number: 10.6084/m9.figshare.21997559 Direct URL to data: https://doi.org/10.6084/m9.figshare.21997559.v2

## Value of the Data

• The most updated dataset on the global distribution of cold-water corals. These vulnerable marine ecosystems provide essential habitat for numerous species, including commercially targeted species, and help regulate carbon sequestration and nutrient cycling. Some of these corals are directly commercially exploited. Yet, they are currently threatened by environmental changes and human activities, such as deep-sea industrialization and fishing.
• The dataset is dereplicated, taxonomically standardized, flagged for potentially incorrect records and provided under the Darwin Core Standard for integration and interoperability.
• It represents a valuable baseline to describe the distribution of species, support biodiversity management and conservation, and address niche-based questions and community changes, including projections of climate-induced distribution range shifts.
• This information is useful for macroecologists, biogeographers and other researchers addressing scientific questions related to cold-water corals, and for conservation biologists guiding stakeholders, resource managers and policymakers for the sustainable use of marine resources, the development of conservation and restoration strategies, and the prevention and mitigation of impacts in these highly vulnerable ecosystems.

## 1 Objective

Complete and accurate data describing the global distribution of species are a prerequisite for studies focused on macroecology, biogeography and conservation [1,2]. Biodiversity data can be obtained from online repositories (e.g., the Global Biodiversity Information Facility [3]); however, these are often incomplete and contain spatial and taxonomic errors [4]. Further, some information is duplicated across databases and is often provided in different formats, precluding proper integration and interoperability. We provide a dataset of cold-water coral distribution records at a global scale, comprising 817,559 dereplicated records of 1170 taxonomically standardized species, integrating a quality control system flagging potentially incorrect records [5]. These data were aggregated from online biodiversity information facilities and peer-reviewed literature and are provided under the FAIR principle of Findability, Accessibility, Interoperability and Reusability and in the Darwin Core Standard [6].

## 2 Data Description

A global distribution dataset of cold-water coral diversity is provided in Excel format, with rows referring to records of occurrence and columns following the data fields of Darwin Core Standard [6] for the location, date and source of records, as well as taxonomy and quality flag of records (Table 1).

**Table 1:** Primary data fields of the dataset of cold-water coral diversity. For information on the additional fields of the dataset please refer to the Darwin Core Standard [6] permanent repository at https://dwc.tdwg.org.

Field	Description
occurrenceID	Identifier assigned to each record
modified	Date-time on which the record was changed
aphiaID	Identifier of the taxon, linked to the World Register of Marine Species
acceptedAphiaID	Identifier of the accepted taxon, linked to the World Register of Marine Species
name	Name of the taxon, as originally reported
acceptedName	Accepted name of the taxon, retrieved from the World Register of Marine Species
status	Status of the taxon (e.g., taxonomically accepted/not accepted)
kingdom	Higher taxonomic classification
phylum	Higher taxonomic classification
class	Higher taxonomic classification
order	Higher taxonomic classification
family	Higher taxonomic classification
genus	Higher taxonomic classification
DecimalLongitude	Geographical longitude in decimal degrees of the record's location
decimalLatitude	Geographical latitude in decimal degrees of the record's location
coordinateUncertaintyInMeters	Distance (in meters) from the decimal Latitude and decimal Longitude that describes the center of the circle containing the record's location
depthAccuracy	Depth uncertainty of the record (in meters), as originally reported
locality	Name of the record's location
verbatimDepth	Depth of the record (in meters), as originally reported
minimumDepthInMeters	Minimum depth of the record (in meters), as originally reported
maximumDepthInMeters	Maximum depth of the record (in meters), as originally reported
year	Four-digit year in which the observation occurred
month	Two-digit month in which the observation occurred
day	Two-digit day in which the observation occurred
originalSourceType	Type of original data source
bibliographicCitation	Bibliographic reference of the record
flagGeographicRange	Flag ‘−1′ for records outside the known distribution range of species
flagLand	Flag ‘−1′ for records over landmasses
flagVerticalRange	Flag ‘−1′ for records outside the known vertical distribution of species
measurementOrFact	Quality control based on the flagging system: flagGeographicRange ‘−1′ for records outside the known geographic distribution of species flagVerticalRange ‘−1′ for records outside the known depth range of species flagLand ‘−1′ for records over land

Initially, biodiversity data were collated from major biodiversity information facilities and literature sources, resulting in 845,712 occurrence records of 1388 species. After taxonomic standardization using the World Register of Marine Species [7], and the deletion of duplicated and non-georeferenced records, the final dataset comprises 817,559 georeferenced records of 1170 taxonomically accepted species, belonging to the orders of Alcyonacea (soft corals), Antipatharia (black corals), Pennatulacea (sea pens), Scleractinia (reef-forming corals), Zoantharia (encrusting or button polyps) of the subphylum Anthozoa, and the order Anthoathecata (athecate hydroids) of the class Hydrozoa. This represents 35% of the estimated number of cold-water coral species [8,9]. The list of the species included in the dataset, their taxonomic rank and the number of records per species are provided in Table S1. Further, the dataset integrates a quality control system, flagging potentially incorrect records using information on the species’ known vertical and geographic distribution. This information was extracted from published literature, databases (e.g., SeaLifeBase [10]), expert range maps (Aquamaps [11] and the International Union for Conservation of Nature [12]) and by expert consulting when possible. A list of the consulted sources is provided in Table S2.

**Fig. 1:**
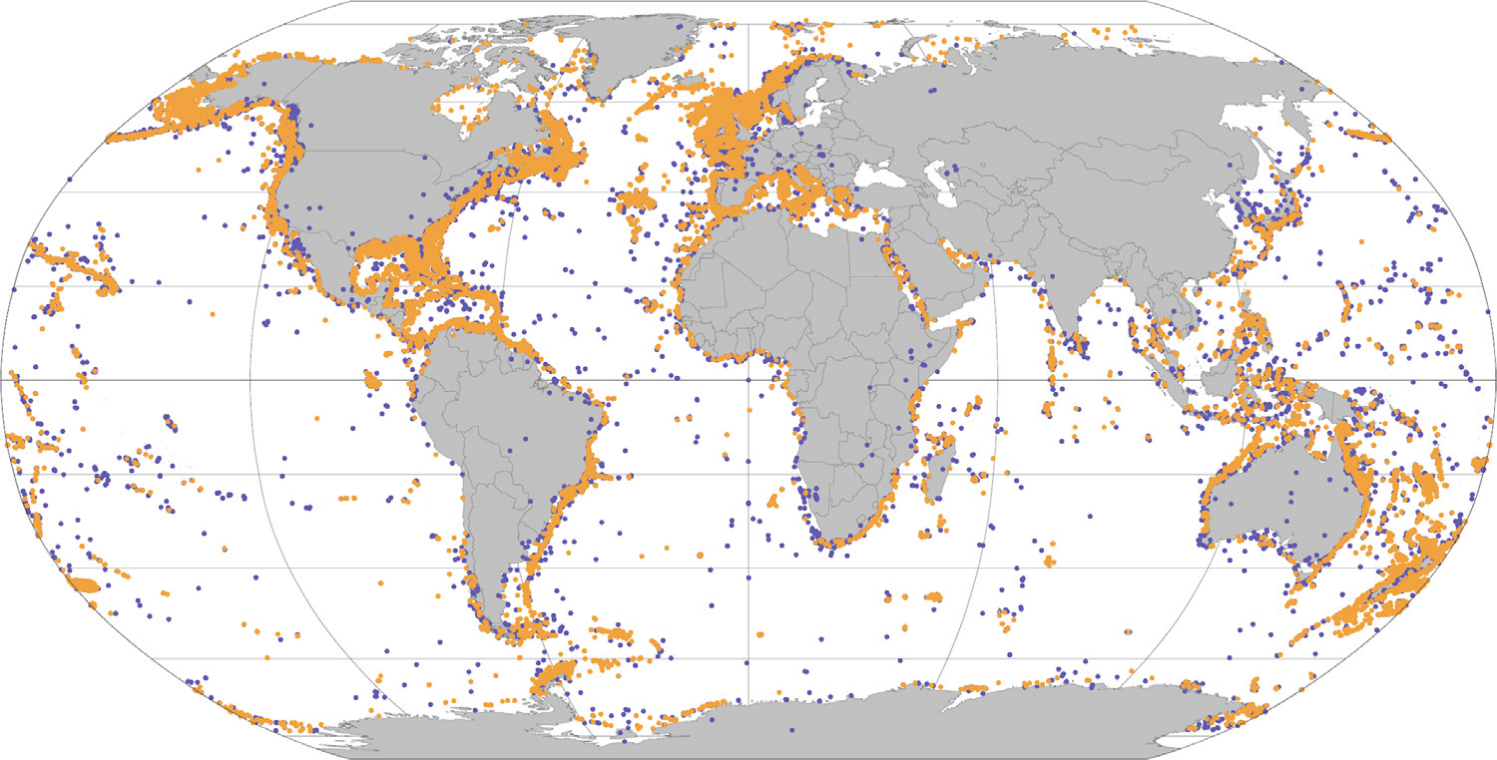
Distribution records of the global dataset of cold-water coral diversity. Purple and orange circles depict flagged and unflagged records, respectively, considering records on land and outside the known geographic and depth distribution of species.

**Fig. 2:**
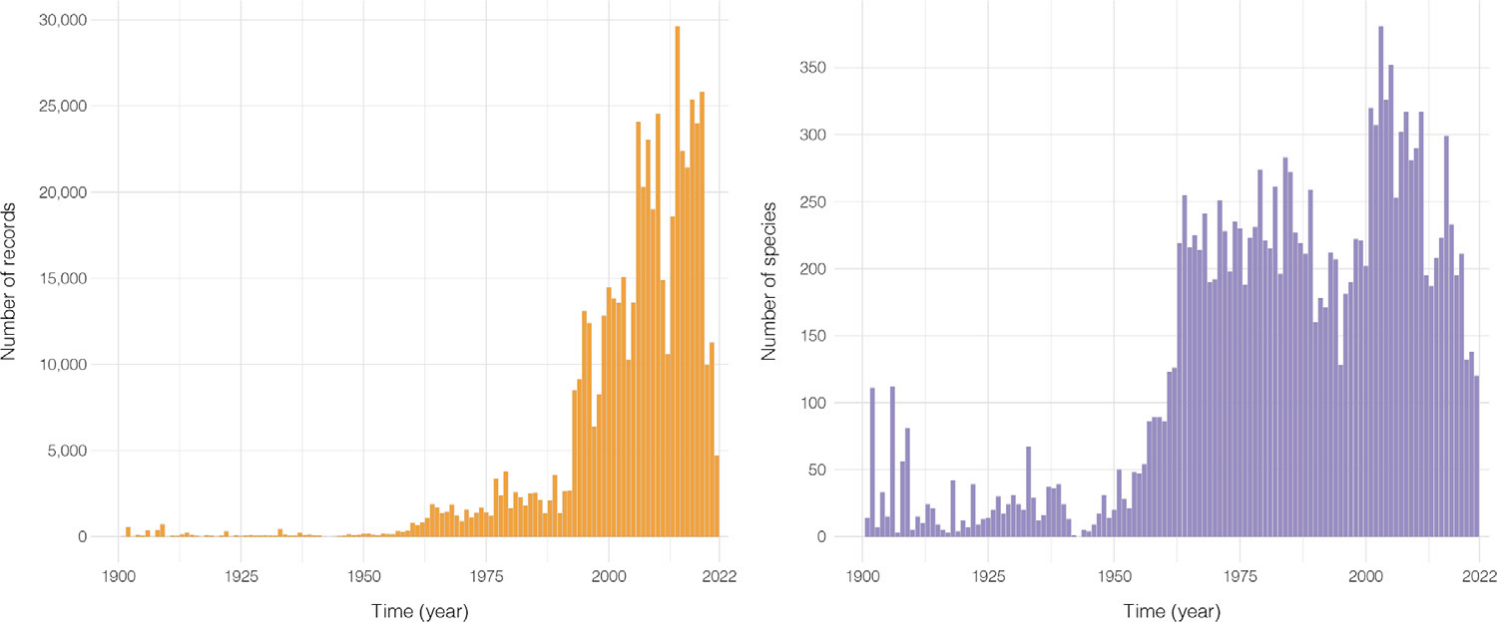
Number of (left panel) records and (right panel) species available in the global dataset of cold-water coral diversity per year (from 1900 to 2022; data are available since the year 1700).

The dataset comprises records spanning across the globe (Fig. 1), from 1700 to 2022 (Fig. 2), and covers ∼80 times more data than the previous baseline dataset “Global distribution of cold-water corals (version 5.1)” of UNEP-WCMC [13] (Table 2).

**Table 2:** Summary of records included in the dataset per higher taxonomic group, original source type and quality flag (considering records on land and outside the known geographic and depth distribution of species). Values in parentheses refer to the percentage of flagged (i.e., identified as likely incorrect) records.

Group	Records (%)	Literature	External databases	Total
Anthozoa	Overall	6108	792,714	798,822
	Flagged: On Land	1265 (20.71)	121,123 (15.28)	122,388 (15.32)
	Flagged: Outside depth range	1220 (19.97)	239,040 (30.15)	240,260 (30.08)
	Flagged: Outside distribution	0 (0)	118,998 (15.01)	118,998 (14.90)
Hydrozoa	Overall	55	18,682	18,737
	Flagged: On Land	0 (0)	2086 (11.16)	2086 (11.13)
	Flagged: Outside depth range	11 (20.00)	3873 (20.73)	3884 (20.73)
	Flagged: Outside distribution	0 (0)	3156 (16.89)	3156 (16.84)
Total	Overall	6163	811,396	817,559

The cold-water coral dataset is publicly available in a permanent repository (https://doi.org/10.6084/m9.figshare.21997559.v2) containing four Excel files:

(1) The flagged final database, containing all occurrence records.
(2) The pruned database, containing only occurrence records flagged as correct based on the known geographic and depth distribution of each species.
(3) Table S1, containing the list of species, their taxonomic rank and the number of records per species.
(4) Table S2, containing the references consulted for the geographic and depth distribution of each species.

## 3 Experimental Design, Materials and Methods

A workflow of cold-water coral data collection and curation is presented below.

### 3.1 Step 1. Importing a List of Cold-Water Coral Species

The taxonomy of corals covers a broad spectrum of species, some of which with tropical affinities outside the scope of the dataset. That means that within the same genus, there are coral species that have either warm or cold climatic affinities. For instance, the genus *Leptogorgia* has numerous species that commonly occur in tropical oceans, at depths shallower than 30 m (e.g., *L. alba* and *L. ignita*), yet *Leptogorgia* species have been also reported in deep, cold environments, like *L. styx* at 2000 m depth [14,15] or *L. cardinalis, L. euryale, L. hebes* and *L. medusa* [16]. Thus, a list of candidate cold-water coral species was obtained from the UNEP-WCMC Global Distribution of Cold-water Corals [13], a dataset that comprises 1261 species of the orders Alcyonacea, Antipatharia, Pennatulacea, Scleractinia, Zoantharia of the subphylum Anthozoa, and the order Anthoathecata of the class Hydrozoa, yet with a limited number of records (10,028 in total).

### 3.2 Step 2. Occurrence Records Collection

Distribution records of the candidate 1261 species were gathered from the following online biodiversity facilities: (1) AquaMaps [11], (2) European Marine Observation and Data Network [17], (3) Global Biodiversity Information Facility [3], (4) Deep Sea Coral Base of the National Oceanic and Atmospheric Administration [18], (5) Ocean Biodiversity Information System [19], (6) The Coral Trait Database [20], (7) Vulnerable Marine EcoSystems Dataset of the International Council for the Exploration of the Sea [21], and from 67 peer-reviewed scientific studies. Information on the original source of each record is provided in the designated data fields of the dataset (Table 1). Only records reporting no copyright and without restrictions for any use or any use with appropriate attribution (e.g., CC0 or CC BY, www.creativecommons.org) were stored in the dataset.

### 3.3 Step 3. Taxonomic Curation

Taxonomic standardization was made for each reported taxon using the World Register of Marine Species (WoRMS) [7]. This authoritative reference system for marine organisms provides a unique identifier (aphiaID), linked to a standardized accepted name and associated taxonomic information. Records with unaccepted status were matched with the currently accepted species name and records with uncertain status were removed from the dataset.

### 3.4 Step 4. Pruning of Occurrence Records

Occurrence records with no coordinate information were removed from the dataset. Further, duplicated records were also removed from the dataset. A record was considered duplicated when belonging to the same taxon and reported in the same coordinates (longitude, latitude and depth) and the same time (year, month and day).

### 3.5 Step 5. Quality Control Flagging of Occurrence Records

The ability to assess the quality of records of large datasets is critical in marine biodiversity research. Regardless of the credibility of the source, incorrect records exist and become shared across data platforms due to automatic interoperability [4]. To overcome this, a quality control system was implemented based on Assis et al., [4] to flag coral records over land mass and with doubtful geographical and depth distributions.

Initially, the depth value of each record was extracted from The General Bathymetric Chart of the Oceans [22], a terrain model for the ocean and land, providing high-resolution depth and altitudinal data (15 arc-second, approx. 465 m at the equator). Records were flagged as over land whenever the extracted bathymetric values were above 0, i.e., referring to altitudinal values. The bathymetric values were further compared to the known vertical distribution of species, as reported in the available peer-reviewed literature, including the sources reported in SeaLifeBase [10] (149 entries; Table S2). Records were flagged as outside depth ranges whenever bathymetric values were higher or lower than the reported range of the corresponding species. Similarly, the geographical location of each record was compared to the known distribution range of species and records were flagged whenever falling outside of it. This procedure was based on distributions obtained from the Food and Agriculture Organization (FAO) Major Fishing Areas [23], which is often used to document species’ distribution ranges, as well as the available peer-reviewed literature (including SeaLifeBase [10]; 311 entries; Table S2), the range maps of Aquamaps [11] and the International Union for Conservation of Nature [12] and by consulting experts whenever possible (290 species in total).

### 3.6 Step 6. Dataset Format Standardization

The final dataset was structured based on the Darwin Core Standard [6], a framework for biodiversity data that offers flexible and stable means to store all fields of original data sources, and provides standard identifiers, definitions and labels. The dataset provided information on the taxonomy, location, date, source and quality flag for each record (Table 1).

### 3.7 Potential Use of the Dataset

The cold-water coral dataset can serve as a valuable baseline to describe species distribution and community composition [24], support biodiversity management and conservation [25], address niche-based questions [26], and understand the relationship between anthropogenic pressures and community changes [27], including predictions of climate-driven distribution range shifts [28] and priority conservation areas with higher or endemic biodiversity [24,28].

## Ethics Statements

The present work complies with ethical requirements and does not involve human subjects, animal experiments, or any data collected from social media platforms.

## CRediT authorship contribution statement

**Viktória Balogh:** Conceptualization, Data curation, Writing – review & editing. **Eliza Fragkopoulou:** Conceptualization, Writing – review & editing. **Ester A. Serrão:** Funding acquisition, Writing – review & editing. **Jorge Assis:** Conceptualization, Data curation, Writing – review & editing, Supervision.

## Declaration of Competing Interest

The authors declare that they have no known competing financial interests or personal relationships that could have appeared to influence the work reported in this paper.

## Data Availability

Global cold-water coral diversity dataset (Original data) (Figshare).
